# Congenital generalized hypertrichosis: the skin as a clue to complex malformation syndromes

**DOI:** 10.1186/s13052-015-0161-3

**Published:** 2015-08-05

**Authors:** Piero Pavone, Andrea D. Praticò, Raffaele Falsaperla, Martino Ruggieri, Marcella Zollino, Giovanni Corsello, Giovanni Neri

**Affiliations:** Unit of Pediatrics and Pediatric Emergency, University Hospital “Policlinico-Vittorio Emanuele”, Catania, Italy; Section of Pediatrics and Child Neuropsychiatry. Department of Clinical and Experimental Medicine, University of Catania, Catania, Italy; Department of Biomedical and Biotechnological Sciences, University of Catania, Catania, Italy; Institute of Medical Genetics, Catholic University, University Hospital A. Gemelli, Rome, Italy; Department of Sciences for Health Promotion and Mother and Child Care, Pediatric Unit, University of Palermo, Palermo, Italy

## Abstract

Hypertrichosis is defined as an excessive growth in body hair beyond the normal variation compared with individuals of the same age, race and sex and affecting areas not predominantly androgen-dependent. The term hirsutism is usually referred to patients, mainly women, who show excessive hair growth with male pattern distribution.

Hypertrichosis is classified according to age of onset (congenital or acquired), extent of distribution (generalized or circumscribed), site involved, and to whether the disorder is isolated or associated with other anomalies. Congenital hypertrichosis is rare and may be an isolated condition of the skin or a component feature of other disorders. Acquired hypertrichosis is more frequent and is secondary to a variety of causes including drug side effects, metabolic and endocrine disorders, cutaneous auto-inflammatory or infectious diseases, malnutrition and anorexia nervosa, and ovarian and adrenal neoplasms. In most cases, hypertrichosis is not an isolated symptom but is associated with other clinical signs including intellective delay, epilepsy or complex body malformations.

A review of congenital generalized hypertrichosis is reported with particular attention given to the disorders where excessive diffuse body hair is a sign indicating the presence of complex malformation syndromes. The clinical course of a patient, previously described, with a 20-year follow-up is reported.

## Introduction

In the normal individual, hair follicles are enclosed in the skin with the exception of the palms, soles, and lips. Hypertrichosis is defined as an excessive growth in body hair beyond the normal variation compared with individuals of the same age, race and sex and affecting areas not predominantly androgen-dependent [1]. The term hirsutism is usually reserved for patients, mainly female, who show extensive hair growth in androgen-dependent areas and with male pattern distribution [2]. The clinical pattern of hypertrichosis can vary in individuals, ranging from lanugo to vellous and terminal hairs. Lanugo refers to long and unmedullated hairs; vellous hairs are unmedullated, short, soft and lightly pigmented and are often seen on the face of infants; terminal hairs are produced by follicles and appear medullated, dense and varying in length, depending on the area [3–7].

Hypertrichosis is classified in different subgroups according to age of onset (congenital or acquired), extent of distribution (generalized or circumscribed), site involved (elbow, neck, lumbosacral region) and to whether isolated or associated with various abnormalities [8, 9].

Acquired generalized hypertrichosis may be secondary to different causes, including drug side effects, metabolic and endocrine disorders (hypothyroidism), dermatomyositis, infectious diseases, malnutrition and anorexia nervosa, and ovarian and adrenal neoplasms [6]. The acquired circumscribed form may be secondary to local pressure, inflammatory lesions, inappropriate use of cosmetics, and to hormonal or steroid stimulation of the skin [7].

The aim of the present study is to offer a clinical survey of generalized congenital hypertrichosis (CGH) and the most common malformation syndromes in which hypertrichosis is a presenting sign and a clue for the diagnosis of the underlying disorders. The clinical course of a patient previously described [10], with an updated follow-up after 20 years is described.

### Historical background

Individuals affected by hypertrichosis have been recorded since the Middle Age and the Renaissance. At that time and in the following centuries, interest was limited to their characteristic phenotype, and they were often exhibited as unnatural phenomena in circuses and fairs due to their peculiar aspect.

Julia Pastrana was a famous woman recognized as affected by this disorder. She was an indigenous Mexican dancer, exhibited in a circus in the United States in the mid-19^th^ century. When she arrived in Europe, Frank Buckland described her as a normal female entirely covered by hair of variable length, sparing her palms and soles [11]. She presented noticeable gingival hyperplasia, a sign that in the following clinical reports would be considered as a feature often associated with hypertrichosis [6, 7].

In the 19^th^ century, the Russian Adrian Jeftichew and his son Fedor, were affected by the same clinical features as the individuals reported above. They presented hypertrichosis on the face and whole body, although the son had less abundant facial hair. In both, hands and feet were spared, together with the anterior portion of the neck and internal face of the arms. In particular, the dentition of Adrian was reported to be partial with several teeth missing [12].

## Epidemiology

The incidence of CGH is unknown, but held to be essentially rare. The incidence is notably higher when the hypertrichosis is one of several signs involved in a complex syndrome. In this case, the incidence of CGH is related to the single condition associated with it [12].

## Etiopathogenesis

CGH in its most common form is idiopathic in the absence of underlying endocrine or metabolic disorders. It is assumed to be related to an excess of stimulation of the hair follicles with normal levels of androgen-like hormones [1]. To date, a clear specific molecular abnormity has not been proved. A hypothesis has been advanced to explain the typical phenotypic aspect of these patients: an atavistic reversion of a suppressed ancestral gene. In the course of evolution genes causing hair growth have been silenced and the appearance of hair in healthy humans can be explained by an erroneous reactivation of such genes [13–15]. In idiopathic CGH, an autosomal dominant trait inheritance with two or more members of the same family affected [16], an X-linked [17] and an autosomal recessive [6, 7] pattern of inheritance have been described. The involvement of chromosome 8 has been reported in patients with generalized congenital hypertrichosis, Ambras type [8, 18, 19], and involvement of chromosome X at the locus Xq24-q27.1 [20] in a family where the males were more affected than the females.

CGH is known to be associated with intellective delay, epilepsy, and malformation features, involving different areas of the body, including facial dysmorphism and abnormalities of eyes, heart, bones and kidneys. Specific molecular defects have been reported in well-known syndromes presenting with hypertrichosis. These include Cornelia De Lange syndrome, with the involvement of genes N11BL, SMC1A, SMC3, RAD21, and HDACE [21]; Coffin-Siris syndrome (BAF complex genes) [22]; Wiedeman-Steiner syndrome (KMT2A gene) [23].

## Diagnosis

Diagnosis of CGH is performed at first glance, but a detailed review of patient history and an in-depth physical examination are necessary to determine the presence of other abnormalities beyond the cutaneous manifestation. Particular attention should be given to the presence of other anomalies in particular the face, eyes, teeth, hearth, kidneys, bones, and extremities, and at the same time, to obesity and intellectual disability. The diagnosis directed at identifying the signs associated with CGH and distinguishing it from the acquired form. The latter group consists of a) drug induced hypertrichosis, in particular anti-convulsants such as phenytoin and other drugs, including corticosteroids, cyclosporine and interferon alpha 2; b) malnutrition and anorexia nervosa; c) endocrine disorders, juvenile dermatomyositis and infectious diseases; d) metabolic diseases such as mucopolysaccharidoses (Hurler, Hunter, Sanfilippo syndromes), congenital porphyrias, and adrenal enzymatic deficiency; e) ovarian and adrenal neoplasms [6]. Depending on the clinical manifestations, laboratory analyses, skeletal X-rays, Brain-MRI, ultrasound EEG, ECG and echocardiogram, and psychometric tests are useful investigations.

## Congenital generalized hypertrichosis: clinical forms

**Fig. 1:**
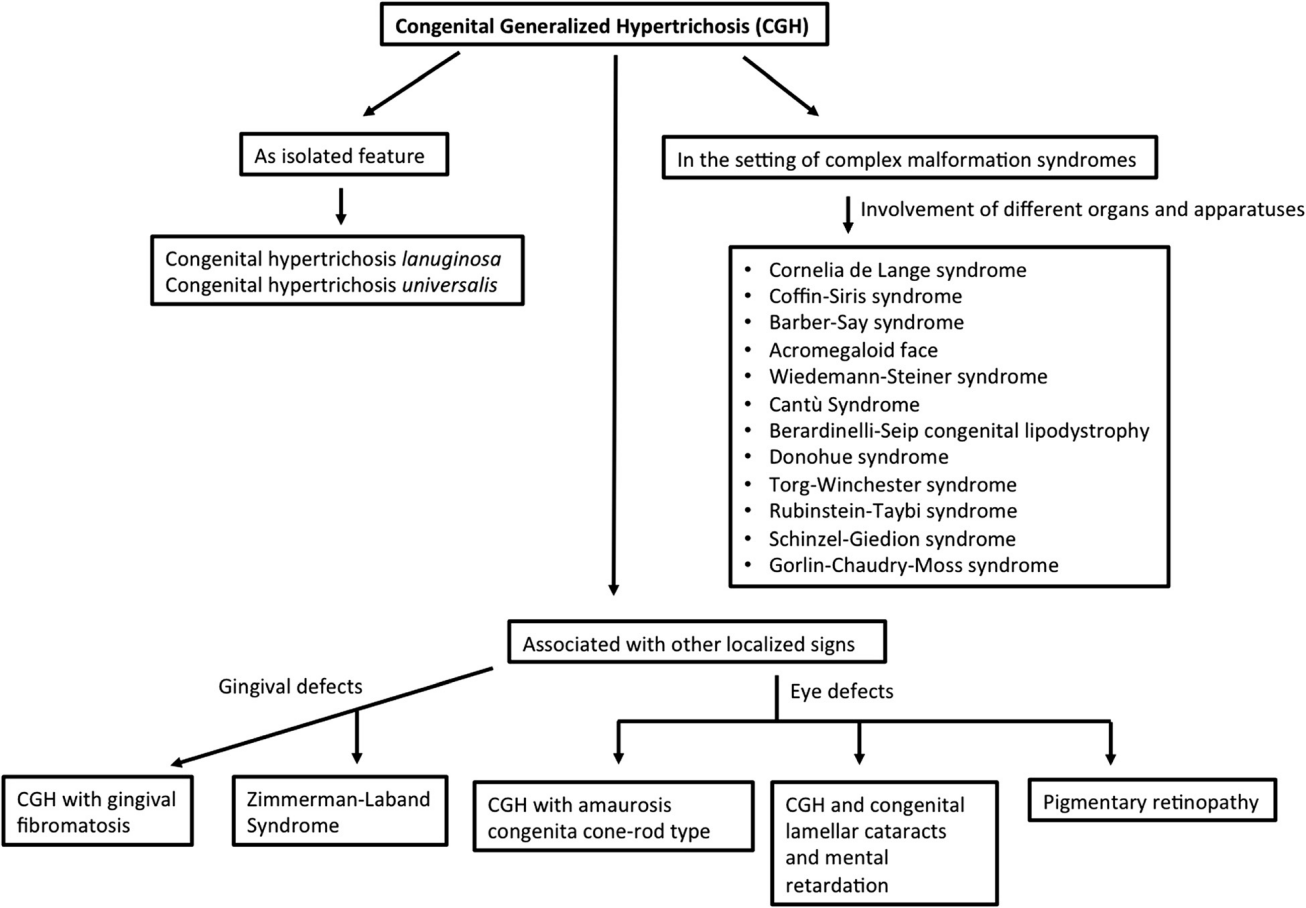
Diagnostic algorithm for Congenital Generalized Hypertrichosis

These forms are characterized by generalized hypertrichosis of the face, trunk and limbs, with very early onset without signs of precocious puberty or virilization. They are often associated with cognitive delay, failure to thrive and signs of dysmorphism of the face and extremities (diagnostic flow-chart in Fig. 1). In this review, we have differentiated CGH as 1) isolated, 2) co-occurring with others anomalies or neurological disorders, 3) in the setting of well-known complex syndromes and 4) disorders with hypertrichosis as an uncommon sign. It should be underlined that some of these forms present common features with each other and some abnormalities could be considered as variants of the same syndrome. These syndromes and their genetic mutations are resembled in Table 1.

**Table 1:** Syndromes presenting with Generalized Hypertrichosis and related genetic mutations. Legend: CGH: congenital generalized hypertrichosis

	Disease	Genes involved (location)
Congenital generalized hypertrichosis as most prominent feature	Congenital hypertrichosis lanuginosa	Inverse mutation on the 8q chromosome
	Congenital hypertrichosis universalis	Autosomal dominant mutation in Xq24-q27.1
Congenital generalized hypertrichosis associated with other anomalies	CGH with gingival fibromatosis	Unknown
	CGH with amaurosis congenita cone-rod type	Unknown
	Cataract, hypertrichosis, and mental retardation (CAHMR) syndrome	Unknown
	Pigmentary retinopathy	Unknown
	Zimmermann-Laband syndrome	Probable breakpoint location in 3p14.3
	Hypertrichosis with coarse face, obesity, short stature and brachydactily	Unknown
Congenital hypertrichosis as a component feature of complex syndromes	Cornelia de Lange syndrome	*NIPBL* (5p13.2); *SMC1A* (Xp11.22-p11.21); *SMC3* (10q25); *RAD21* (8q24.11); *HDAC8* (Xq13)
	Coffin-Siris syndrome	*BAF* complex genes (19p13.2; 22q11.23; 9p24.3; 17q21.2; 1p36.11; 6q25.3)
	Barber-Say syndrome	*KMT2A* (11q23)
	Acromegaloid facial appearance with hypertrichosis	*PGM1* (1p22.1); *GLO1* (6p21.3-p21.1), *IGHG3* (14q32.33), and *HP* (16q22.2) [low positive LOD scores for linkage]
	Wiedemann-Steiner syndrome	*KMT2A* (11q23)
	Osteocondrodysplasia with hypertrichosis or Cantù Syndrome	*ABCC9*; *KCNJ8* (12p12.1)
	Berardinelli-Seip congenital lipodystrophy	*BSCL* (11q13)
	Donohue syndrome	*INSR* (19p13.2)
	Torg-Winchester syndrome and nodulosis arthropathy-osteolysis	*MMP2* (16q13-q21)
	Rubinstein-Taybi syndrome	*CREBBP* (16p13.3); *EP300* (22q13.2)
	Schinzel-Giedion syndrome	*SETBP1* (18q21.1)
	Gorlin-Chaudry-Moss syndrome	Unknown
Disorders with congenital generalized hypertrichosis as an uncommon feature	Hemi-maxillo facial dysplasia	Unknown
	Craniofacial dysostosis	Unknown
	Hypomelanosis of Ito	Probable breakpoint location in Xp11

### CGH as most prominent feature

#### Congenital hypertrichosis lanuginosa (CHL)

CHL is characterized by the presence of fine, blond hair of lanugo type, distributed all over the body except for the palms, soles and mucous membranes [6, 11]. In normal individuals, lanugo hairs are often present at birth and tend to gradually disappear over the first months of life. In affected patients, the lanugo distribution continues to progress, involving the whole body [7]. The disorder is also known as “Ambras Syndrome” referring to the painting of Peter Gonzales kept in Ambras Castle, Austria [11]. This form has been reported in patients with abnormalities of chromosome 8q [8, 18] and as autosomal dominant trait inheritance but also as sporadic events. [7].

#### Congenital hypertrichosis universalis

In this disorder, patients show a clinical presentation similar to CHL, with hairs distributed over the face, trunk, back and limbs. The individuals affected present with terminal hairs from birth. Familial cases have been reported with autosomal dominant inheritance and X-linked with a responsible gene mapped to Xq24-q27.1 [20].

### CGH associated with other anomalies

#### CGH with gingival fibromatosis

In this form, alongside hypertrichosis, patients present with gingival overgrowth, which is not related to any drug treatment, including anticonvulsants and cyclosporine [24]. In addition, the affected patients may present cognitive delay and/or epilepsy [25–27]. Douzgou reported a typical example of a patient with all those anomalies [28]. The gingival overgrowth may be diagnosed later. Patients with *de novo* mutation and also with autosomal recessive inheritance have been reported [29].

#### CGH with amaurosis congenita cone-rod type

A large family with an autosomal recessive inheritance was reported by Jalili. [30]. The affected patients present a clinical pattern of ocular anomalies, with Leber-type retinal involvement, and cone-rod dystrophy. In addition, the patients show trichomegaly, bushy eyebrows and synophrys.

#### CAHMR syndrome

CGH has been also associated with congenital lamellar cataracts and mental retardation, with the acronym CAHMR syndrome. Additional features consist of macrodontia and pectus excavatum. In a family, an autosomal recessive inheritance was suggested [31].

#### Pigmentary retinopathy

In 1996, Pivnick et al reported on a 22-month-old child who showed diffuse hypertrichosis, facial structural anomalies, symmetrical hyperpigmentation of the sideburn areas of the face and hyperpigmented streaks on the limbs, dolicocephaly and pigmentary retinopathy. The hyperpigmented skin showed histological features of many separate bundles of smooth muscles in the derma [32].

#### Zimmermann-Laband syndrome

The syndrome is characterized by gingival hypertrophy, hypo/aplastic nails and distal phalanges, hypertrichosis and intellectual disability. Hypertrichosis is a presenting sign. Molecular anomalies are not known. An autosomal dominant with high mutation rate and rare instances of germinal mosaicism are reported as expression of inheritance [33].

#### Hypertrichosis with coarse face, obesity, short stature and brachydactily

In 1996, some of the authors of the present article, described a patient who showed CGH, coarse facial features, obesity and, moreover, short stature, brachydactily with broad proximal phalanges and small, dysmorphic nails [10].

### Congenital hypertrichosis as a component feature of complex syndromes

#### Cornelia de Lange syndrome

In this syndrome, generalized hypertrichosis is present together with cutis marmorata in 60 % of patients; the cutaneous signs are small nipples and umbilicus and premature aging signs (wrinkling, sagging and gray hair) [34]. Microcephaly, short neck, and a characteristic facies consisting in low anterior and posterior hairlines, arched eyebrows and synophrys, narrow palpebral fissures and long, curly eyelashes are the presenting signs of the syndrome. Thick, low set and posteriorly rotated ears, flat midface, short broad nose and depressed nasal bridge with anteverted nares, down-turned corners of the mouth and micrognathia are often observed. High and cleft palate (often submucous) and dental anomalies may be present. Patients affected by this syndrome often show prenatal and postnatal growth retardation, short stature with short hands and feet and psychomotor delay. Other signs are usually present: hearing loss, scoliosis, cervical malformations and pectus excavatum, early osteoporosis and pyloric stenosis. In this syndrome, a 25-30 % incidence of congenital heart disease, 40 % of structural kidney and/or urinary tract anomalies, 73 % cryptorchidism and 57 % hypoplastic genitalia have been reported [35]. Mutations in *NIPBL, SMC1A, SMC3, RAD21* and *HDAC8* genes have been linked with this syndrome [21].

#### Coffin-Siris syndrome

Patients with Coffin-Siris syndrome show signs of hypertrichosis, sparse scalp hair, bushy eyebrows and eyelashes. There is almost always an abnormal or delayed dentition, together with ear anomalies and absence of the distal phalanx and nails of the fifth fingers and toes. Mutations in BAF complex genes are the main cause of the syndrome [22, 36, 37].

#### Barber-Say syndrome

Aside hypertrichosis, patient with Barber-Say syndrome present atrophic lax skin, macrostomia, broad and coarse eyebrows, an hairy bulbous nasal tip with hypoplastic flaring nostrils, telecanthus and hypoplastic nipples. Mutations in *KMT2A* gene have been found to be the cause of the disorder [38–40].

#### Acromegaloid facial appearance with hypertrichosis

This rare syndrome is characterized by hypertrichosis terminalis, coarse facies, bulbous nose, thickened lips, narrow palpebral fissures, thick intraoral mucosa, high arched eyebrows, mental retardation (not always present), large hands, hyperextensible joints, recurrent pericardial effusions and hypotestosteronemia. Autosomal recessive inheritance has been suggested [41, 42].

#### Wiedemann-Steiner syndrome

Hypertrichosis and prominent forehead with low anterior and posterior hairlines, thick eyebrows, hypertelorism, wide nasal bridge and small ears are the main features of the syndrome. Patients also show hypotonia, short stature, markedly advanced bone age, hypoplastic 12th ribs and a dysplastic hip, doughy and redundant skin on hands and hypoplastic middle phalanx of the fifth finger. Mutations in *KMT2A* gene complex have been found in the patients affected [23, 43].

#### Osteocondrodysplasia with hypertrichosis or Cantù Syndrome

Patients show neonatal macrosomia, with wide posterior fossa in the skull, a distinctive osteocondrodysplasia, coarse face, cognitive delay and cardiomegaly with cardiomyopathy. A mutation in the *ABCC9* or with *KCNJ8* gene has been reported [44–46]. The bone involvement is complex with narrow thorax, broad ribs, platyspondyly, hypoplastic idiopathic branches, small obturator foramen, bilateral coxa valga, large medullary canals and generalized osteopenia. Short distal phalanx of the thumbs and of the first toes, delayed bone age and hypertrophy of the first metatarsus are also found.

#### Berardinelli-Seip congenital lipodystrophy

This syndrome is an autosomal recessive disorder with various dermatological features and systemic manifestations. Types 1 and 2 have been distinguished, with the latter more common and severe, with onset in the neonatal period or in early infancy [47]. Lipoatrophic diabetes and generalized congenital hypertrichosis are the main features of the syndrome. Patients present with a distinctive facial appearance with sunken cheeks, large ears, curly scalp hair and external genital hypertrophy [48–50]. Diabetes is insulin-resistant without ketosis. In this disorder, the hair tends to become more pronounced with age. Cognitive delay, corneal opacities, hepatomegaly and cardiac renal abnormalities are reported [49, 50]. Other dermatologic features consist of acanthosis nigricans, prominent subcutaneous veins and xantomas. The locus for *BSCL* has been identified in chromosome 11q13 [51].

#### Donohue syndrome (DS)

Also known as leprechaunism, DS is a severe form of congenital insulin resistance, due to a mutation in the insulin receptor gene. It is characterized by dwarfism with a peculiar elfin-like face, large eyes, thick lips and low set ears. Patients present with metabolic abnormalities and increased levels of androgens, together with loss of subcutaneous fat with excessive folding of the skin [52]. Hypertrophy of external genitalia, abdominal distension and slow growth may also be present.

Donohue syndrome shows features similar to Berardinelli Seip syndrome but its course is more severe and insulin resistance is more precocious. The increased early mortality is linked to anomalous energetic metabolism and loss of glucose homeostasis. Tentative treatment with recombinant human IGF1 has been started [52].

#### Torg-Winchester syndrome and nodulosis arthropathy-osteolysis (NAO)

Nosographic delineation of these syndromes has not been defined. Clinical manifestations include dwarfism, with joint and bony abnormalities, peripheral corneal opacities, and coarse facial features [53]. Localized thickened cutaneous plaques are reported. Bone abnormalities consist of progressive osteolysis affecting the carpal, tarsal and interphalangeal joints. Involvement of metalloproteinasis2 gene (*MMP2* gene) has been reported [54–60].

#### Rubinstein-Taybi syndrome (RTS)

RTS is an autosomal dominant disorder characterized by typical features of bird-like facies and hypertelorism, microcephaly, broad thumbs and big toes, cognitive delay and postnatal growth retardation. Mutations in the genes encoding the cyclic-AMP regulated enhanced binding protein (CREBBP) and the E1A binding protein p300 (EP300) have been found in patients with this syndrome [57–59].

#### Schinzel-Giedion syndrome (SGS)

The syndrome is characterized by multiple congenital anomalies with craniofacial dysmorphism (midfacial retraction, protruding forehead) and severe cognitive delay. The neck is short, with abundant folds of skin. The gene involved in this disorder is SETBP1. The syndrome has been associated with various neurological abnormalities, including cobblestone lissencephaly and epileptic seizures (West syndrome type). Radiological bone abnormalities affect the skull, ribs and terminal phalanxes [60, 61].

#### Gorlin-Chaudry-Moss syndrome

This congenital malformation syndrome is characterized by craniofacial dysostosis (mid-facial flattening), underdeveloped genitalia, patent ductus arteriosus and hypoplasia of teeth, and digital defects. To date, no pathological deletions or duplications have been found [62, 63].

### Disorders with CGH as an uncommon feature

#### Hemi-maxillo-facial dysplasia

In this disorder the face is asymmetric with unilateral maxillar enlargement and with ipsilateral facial hypertrichosis. The teeth are hypoplastic [7].

#### Craniofacial dysostosis

Is often associated with cardiac anomalies, hypoplasia of the labia majora, together with abnormalities of the teeth and eyes [7].

#### Hypomelanosis of Ito

Is a complex cutaneous disorder characterized by hypopigmented macular patches following the Blaschko lines frequently associated with neurological impairment such as cognitive delay and epilepsy. In one patient, the normal skin presented with hypertrichosis while the hypomelanotic areas did not [7, 64].

## Prognosis

As discussed above, congenital generalized hypertrichosis is a clinical sign of different disorders. The distribution of hair is usually precocious and rarely progressive. The prognosis is related to the pathological events associated with the hair disorder, in particular to the epilepsy and cardiac or renal anomalies.

## Management and treatment of hypertrichosis

Congenital generalized hypertrichosis is cause of significant emotional distress for the affected patients and their family. The cosmetic embarrassment is particularly relevant when the hair is widely distributed over the areas of the body normally uncovered. There are different approaches to the treatment of excess hair, including:

1. cosmetic procedures
2. intense pulsed light source and laser treatment
3. pharmacological treatment

It should be underlined that not all the treatments are effective over the long term, and the choice of therapy should be made taking into consideration such aspects as the location of the excess hair, its association with complex anomalies and the age of the patients.

Cosmetic procedures consist of using bleaching methods to make dark-colored hair less evident or of using different procedures to remove the excess hair, such as trimming, shaving, plucking or waxing. Clinical depilation acts by damaging the hair directly on the skin surface, while electrosurgical epilation, using a fine electric needle inserted into the hair follicle, is more effective.

Important results are obtained by intense pulsed light sources and various type of lasers (Ruby, Alexandrite, Diode). In particular, a Neodynium:Yttrium-Albumin-Garnet (Nd:YAG) laser has been used with appreciable results in the removal of excess hair [65].

Pharmacological treatment consists of topical eflorinithine, a specific and irreversible inhibitor of the enzyme ornithine decarboxylase, which is located within the hair follicle and stimulates hair growth [6, 66].

When present in the setting of systemic disorders, hypertrichosis treatment is usually linked to the associated anomalies. Cosmetic treatment is always useful to avoid the social rejection and isolation of patients. The use of shaving or clinical hair removal may give excellent results but it may also cause irritation and allergic contact dermatitis. Good results can be achieved with electrolysis or Alexandrite laser, but repeated sessions are usually required. In some cases, depilation may be definitive. In one third of cases regrowth of the hair may occur, thus necessitating further procedures.

## Conclusions

Generalized congenital hypertrichosis is a wide topic in the field of pediatric practice. Generalized congenital hypertrichosis is not only a cause of cosmetic embarrassment but also a clue to various complex syndromes.

Although this clinical event is rare, it can cover numerous conditions, which can create genetic and prognostic problems not only in the patient but in subsequent progeny. In particular, the severe consequences of these disorders necessitate a major effort to better define the clinical focus and the pathogenesis and to develop pharmacological treatments aimed at making this pathology less unpleasant.

## Addendum

A 17-year-old male with hypertrichosis, coarse facial features, brachydactily, obesity and intellectual delay was reported by our group in 1996 [10]. The patient showed generalized hypertrichosis of terminal hair from birth. The hair was distributed along the body with a whorl-shaped pattern in the middle of the back and the eyebrows were thick. The face was round and coarse. The hands and feet were small, the proximal phalanges broad and the nails small and dyschromic. Intellectual delay was present.

We have periodically followed up this patient since the age of 17 years for further 19 years. His general condition has not changed. At the age of 20 years, he started to present frequent episodes of generalized tonic-clonic seizures, partially controlled by anticonvulsants (carbamazepine) and for some years by levetiracetam with better results. Intellective delay is moderate-severe. He is not aggressive, lives at home but attends an institution for intellective delay. He shaves twice a day. He was advised to follow a diet, with poor compliance, and is still overweight.
